# Avian haemosporidian persistence and co-infection in great tits at the individual level

**DOI:** 10.1186/1475-2875-12-40

**Published:** 2013-01-30

**Authors:** Juan van Rooyen, Fabrice Lalubin, Olivier Glaizot, Philippe Christe

**Affiliations:** 1 Department of Ecology and Evolution, University of Lausanne, CH-1015 Lausanne, Switzerland; 2, Museum of Zoology, CH-1014 Lausanne, Switzerland

**Keywords:** *Plasmodium*, *Haemoproteus*, *Leucocytozoon*, Multiple infection, Competition

## Abstract

**Background:**

Many studies have tracked the distribution and persistence of avian haemosporidian communities across space and time at the population level, but few studies have investigated these aspects of infection at the individual level over time. Important aspects of parasite infection at the individual level can be missed if only trends at the population level are studied. This study aimed to determine how persistent Haemosporida are in great tit individuals recaptured over several years, whether parasitaemia differed by parasite lineage (mitochondrial cytochrome b haplotype) and how co-infection (i.e. concurrent infection with multiple genera of parasites) affects parasitaemia and body mass.

**Methods:**

Parasite prevalence was determined by polymerase chain reaction (PCR), quantitative PCR were used to assess parasitaemia and sequencing was employed to determine the identity of the lineages using the MalAvi database.

**Results:**

Haemosporidian prevalence was high over sampled years with 98% of 55 recaptured individuals showing infection in at least one year of capture. Eighty-two percent of all positive individuals suffered co-infection, with an overall haemosporidian lineage diversity of seventeen. *Plasmodium* and *Haemoproteus* parasites were found to be highly persistent, with lineages from these genera consistently found in individuals across years and with no differences in individual parasitaemia being recorded at subsequent captures. Conversely, *Leucocytozoon* parasites showed higher turnover with regard to lineage changes or transitions in infection status (infected *vs* non-infected) across years. Parasitaemia was found to be lineage specific and there was no relationship between *Plasmodium* parasitaemia or host body condition and the presence of *Leucocytozoon* parasites.

**Conclusions:**

The findings of this study suggest that different genera of haemosporidian parasites interact differently with their host and other co-infecting parasites, influencing parasite persistence most likely through inter-parasite competition or host-parasite immune interactions. Even-though co-infections do not seem to result in increased virulence (higher parasitaemia or poorer host body condition), further investigation into infection potential of these parasites, both individually and as co-infections, is necessary.

## Background

Haemosporidians are well-known and extensively studied parasites as *Plasmodium* gives rise to malaria in humans and animals, remaining one of the most common diseases in warm climate countries [1]. Investigating interactions between hosts and their parasites as well as the factors governing host susceptibility is key for understanding the epidemiology of the disease and host-parasite coevolution.

The importance of avian haemosporidian parasites (*Plasmodium* sp., *Haemoproteus* sp. and *Leucocytozoon* sp.) as a model system for studying host-parasite evolution and the consequences on ecology and conservation has been increasing over recent decades [2]. A number of studies have shown the costs on life-history traits associated with haemosporidian infection. Avian haemosporidian parasites can affect host body condition [3], reproductive success [4-6] and survival [7-10], with extreme cases resulting in the extinction of the avian host [11-13]. Consequently, these parasites can exert strong selective forces on their hosts.

As avian heamosporidians are ubiquitous [8], birds are exposed to a variety of haemosporidian parasites [8,14-21] and the distribution of these blood parasites within and between host populations has the potential to reveal different evolutionary dynamics of host-parasite interactions [22]. Therefore, knowledge of the persistence of parasite communities and their composition across temporal scales is a prerequisite for investigating and determining these host-parasite interactions.

Many studies have investigated haemosporidian community composition across space and time at the population level [22-25] and found that most parasite communities remained stable (but see [23]), even for up to 17 years [22]. However, few studies have investigated haemosporidian communities at the individual level. When studying communities at the population level only, changes happening at the individual level might be overlooked, especially if these changes have fast turn-over. Therefore, while parasite communities at the population level appear stable, at the individual level hosts might be experiencing rapid changes in parasite onslaught. At the individual level Hasselquist *et al*[25] found that the likelihood of retaining the same infection status (infected vs. uninfected) for *Haemoproteus payevskyi* was higher than the probability of experiencing a change in status. Knowles *et al*[26] showed that 26% of *Plasmodium* infection in individual blue tits (*Cyanistes caeruleus*) from the UK could be lost over time, Piersma and van der Velde [27] found that 23% of house martins (*Delichon urbicum*) in the Netherlands showed no haemosporidian infection status changes over time and Latta and Ricklefs [28] found high individual turn-over in haemosporidian infections between years in various host species on the island of Hispaniola.

While a number of factors can influence the persistence and abundance of a parasite in a host (e.g. host health or immunocompetence [29,30] and environmental condition [31]), the presence of multiple parasites within a host, i.e. co-infection, can critically impact infection dynamics and virulence. Co-infections with different haemosporidian genera can result in within-host competition leading to increased virulence [32]. On this topic, studies of mixed infections in birds have yielded diverse results. Palinauskas *et al*[33] reported increased virulence in experimentally co-infected individuals although this effect was host-species specific. Conversely, Marzal *et al*[34] showed that co-infection results in increased mortality but higher reproductive success in house martins, presumably as a result of increased investment in reproduction. Finally, Davidar and Morton [35] revealed that although single infections of *Haemoproteus prognei* and filarial nematodes are relatively harmless in purple martins (*Progne subis*), a co-infection of these two parasites almost exclusively result in death of the host.

This study reports data from wild, free-living great tits (*Parus major*), sampled across a three-year period and addresses the following questions: 1) how persistent are the three haemosporidian genera (*Plasmodium*, *Haemoproteus* and *Leucocytozoon*)? 2) Are there lineage-specific (a lineage being defined as a *Cyt b* haplotype) differences in parasitaemia? and 3) what is the frequency of co-infections and do co-infections between *Leucocytozoon* and *Plasmodium* appear to impact *Plasmodium* parasitaemia and host body condition? Taken together, these data will enable a better understanding of the dynamics of parasite infection.

## Methods

### Great tit captures and handling

A total of 311 nestboxes were installed in temperate broadleaf, mixed forests throughout the Canton of Vaud in western Switzerland. Adult great tits (*Parus major*) were trapped in their nestboxes during three consecutive breeding seasons (2009-2011) by using door traps mounted inside the nestboxes when their nestlings were twelve days old. Body mass was measured to the nearest 0.1 g. A 30 *μ*l blood sample was taken by brachial venapuncture, and collected in lithium-heparin lined Microvettes ^*â“‡*^ (CB 300 LH, Sarstedt, Germany). Only birds captured in more than one season were considered for this study (n = 55). All birds were captured under license from the Swiss Federal Office for the Environment (number F044-0799), and in accordance with the Cantonal Veterinary Authorities of the Canton de Vaud, Switzerland (authorization number 1730).

### Molecular analyses

DNA was extracted from blood using the DNeasy tissue extraction kit (QIAGEN) according to the manufacturer’s protocol for purification of DNA from blood using the BioSprint 96. After DNA extraction a nested PCR refined by Waldenström *et al*[36] from the original protocol made by Bensch *et al*[14] was performed on all samples. The full method is described in [37] with the following modifications: The PCR cycle profile included an initial denaturation at 94°C for 3 minutes followed by 20 cycles of 94°C for 30 s; 50°C for 30 s; 72°C for 45 s, and with a final extension at 72°C for 10 min. The final PCR amplification was run for 35 cycles with the same thermal profile as described here. For *Leucocytozoon* amplification, initial primers HaemNFI and HaemNR3 and nested primers HaemFL and HaemR2L (amplifying a 480 bp *Cyt b* fragment) were used [38] with the same thermal profile and procedure as described. Reactions were run on a Veriti 96 Well Thermal Cycler (Applied Biosystems). Distilled RNAse-free water was used as a negative control and one negative control was inserted for every nine samples tested. Genomic DNA from individuals with known malarial infections were used as positive control. Nested PCR products were separated on 2% agarose gels containing ethidium bromide and a sample deemed positive if a fragment of approximately 500 bp was present when the gel was viewed under a UV light.

Nested PCR products were purified using the Wizard SV Gel and PCR Clean-Up System (Promega) using the manufacturer’s protocol for DNA purification by centrifugation. Purified PCR products were sequenced in both directions using the primers HaemF and HaemR2 for *Plasmodium* and *Haemoproteus* and primers HaemFL and HaemR2L for *Leucocytozoon* with a dye terminator cycle sequencing (BigDye ^*â“‡*^ v3.1) reaction and electrophoresis was carried out on an ABI Prism 3100 sequencer (Perkin Elmer, Norwalk, CT). The sequences were assembled and edited using CodonCode Aligner (CodonCode Corporation). Sequences were then identified by performing a local BLAST search with the MalAvi database [2]. All *Plasmodium* and *Haemoproteus* sequences could be identified (or characterised as multiple infections), while 3.5% of *Leucocytozoon* samples were unidentifiable after sequencing due to poor DNA quality resulting in unreadable chromatographs. Similarly, identification of lineages involved in multiple infections were not possible (38.9% of infected birds suffered multiple *Leucocytozoon* infections, while 3.7% suffered multiple *Plasmodium* / *Haemoproteus* infections).

*Plasmodium* and *Haemoproteus* parasite quantification was achieved using a real-time quantitative PCR (qPCR) assay described in [37] but using a parasite *Cyt b* TaqMan probe (CY3-CYTb-BHQ2: 5’-CCTTTAGGGTATGATACAGC-3’) and a host 18S rRNA probe (FAM-18S-BHQ1: 5’-AACCTCGAGCCGATCGCACG-3’), and calculated in the following manner: Firstly, parasite and host DNA quantity present in a sample was calculated by the equation

(1) $$\alpha =10^{\frac{1}{m(△\text{Ct})}}$$

Where *α* represents the DNA quantity in the sample; m represents the slope of the standard curve; and △Ct is the difference between the mean Ct value of the sample and the intercept of the standard curve. Ct (or threshold cycle) is the fractional cycle number at which the fluorescence is detected to significantly surpass the threshold. This equation then gives a relative quantification value for parasitaemia relative to a standard sample used. Relative parasite density relative to host (R *α*) was then calculated by the equation

(2) $$\mathrm{R\alpha}=\frac{\alpha (\text{parasite})}{\alpha (\text{host})}$$

As a result of the exponential relationship between Ct and R *α*, the log_10_ of R *α* was used to linearize this relationship. Parasitaemia is, therefore, unitless. The same reference sample was used for all qPCR standard curves and the efficiency of each qPCR run is taken into account during the calculation, therefore, parasitaemia values for samples collected in different years are directly comparable.

### Cloning of multiple infections

Cloning was performed on a randomly chosen subset of ten samples containing multiple double peaks when DNA chromatographs were viewed. This was done to verify that the double peaks observed indicated multiple infections of parasites within a single host or due to problems like amplification errors arising from sequencing. Cloning was performed using the pGEM ^*â“‡*^-T Easy Vector System (Promega) according to the manufacturer’s protocol. Ligation reactions were set up using purified PCR products and cultures of transformed high efficiency competent cells were plated onto LB/ampicillin/IPTG/X-Gal plates. Eight positive colonies were selected at random from every cloned sample. Inserts of positive colonies were sequenced using the SP6 and T7 Promoter Primers (Promega). Due to the cloning procedure being prone to introducing artificial mutations, only lineages that were detected in two or more separate colonies from each sample were considered as lineages involved in co-infection. Cloning confirmed that the double peaks on DNA chromatographs indicated infection with multiple lineages at the same time within a single host.

### Statistical analyses

The analyses were performed with the freeware R-Cran Project [39]. A Fisher’s exact test was performed to determine differences in the frequency of lineage identity changes and infection status (infected vs. uninfected) changes between *Plasmodium*, *Haemoproteus* and *Leucocytozoon* infections in recaptured individuals. Parasitaemia data were log transformed to achieve normality. A Welch two-sample t-test was used to assess the difference in parasitaemia between the two most prevalent *Plasmodium* lineages, SW2 (*P. polare*) and SGS1 (*P. relictum*), as sample sizes for other recorded lineages were too low to be considered. Paired t-tests were performed to determine whether an individual’s parasitaemia changed between years of capture. Linear mixed models (lmer) in the lme4 package [40] were performed on parasitaemia data to determine whether there is a relationship between concurrent infection with *Leucocytozoon* and *Plasmodium* parasitaemia. *Plasmodium* parasitaemia was considered as response variable with bird identity and year of capture as random factors and *Leucocytozoon* infection as fixed effect. To assess the virulence (i.e. affect on host body mass) of co-infection, linear mixed models were performed on a subset of data containing only occurrences of lineage SGS1 (n = 83) and considering *Leucocytozoon* infections as presence or absence values only. Body mass was considered as response variable with bird identity as random factor and sex and *Leucocytozoon* infection as fixed effects. Likelihood ratio tests (LRT) were used to compare “goodness of fit” between models containing and excluding the fixed effect of interest. Probability values of *p* ≤ 0.05 were considered significant.

## Results

### Great tit recaptures

A total of 328 adult great tits (*Parus major*) were captured over the three breeding seasons (110 great tits in 2009, 158 in 2010 and 60 in 2011). Amongst them, 55 birds were recaptured in one or both subsequent years to their first capture. In 2010 there was a recapture rate of 22.8% (n = 158) (i.e. birds caught in 2010 that were also trapped the previous year) and a recapture rate of 38.3% (n = 60) in 2011 (birds that were previously trapped in 2009 and/or 2010).

### Parasite prevalence, persistence and parasitaemia

**Figure 1:**
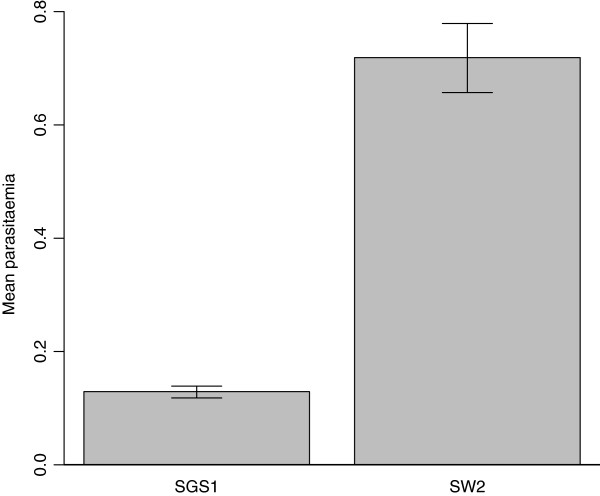
***Plasmodium *parasitaemia.** Parasitaemia (with standard error) of the two most prevalent Plasmodium lineages - SGS1 (Plasmodium relictum, n = 83) and SW2 (Plasmodium polare, n = 9). Individuals infected with SW2 showed higher parasitaemia than individuals infected with SGS1 (t_(13.17)_=−2.84, p <0.05).

Fifty-four (98.2%) of all recaptured great tits tested positive for haemosporidian infection in at least one year of capture. A total of three *Plasmodium*, two *Haemoproteus* and 13 *Leucocytozoon* lineages were identified (Tables 1 and 2). Of these, eight *Leucocytozoon* lineages were detected which were previously unencountered in the literature, and these new lineages differed by 2 - 8% from PARUS22, which was the most prevalent and widespread *Leucocytozoon* lineage encountered. *Plasmodium* and *Haemoproteus* parasites were more persistent (i.t.o. infection status and lineage identity changes) than *Leucocytozoon* parasites (*p* < 0.0001, Fisher’s exact test) indicating that host-parasite interactions between great tits and *Leucocytozoon* parasites and parasite-parasite interactions between *Leucocytozoon* parasites are more dynamic than *Plasmodium* and *Haemoproteus* host-parasite and parasite-parasite interactions. Ninety-three percent of birds remained infected with the same *Plasmodium* or *Haemoproteus* lineage at subsequent capture, while only 43.8% of birds retained the same *Leucocytozoon* lineage. Parasitaemia of *Plasmodium* infection was dependent on the lineage with which an individual was infected. Individuals infected with lineage SW2 (*Plasmodium polare*) showed higher parasitaemia than individuals infected with SGS1 (*Plasmodium relictum*) (Figure 1). An individual’s parasitaemia did not change from one capture to a subsequent capture (paired t-test: 2009-2010: t_(24)_=−0.27, *p* =0.791; 2010-2011: t_(11)_=−0.78, *p* =0.450; 2009-2011: t_(3)_=−2.25, *p* =0.110).

**Table 1:** Haemosporidian lineage observations

Lineage	GenBank accession no.	Morphospecies	***n***
SGS1	AF495571	*Plasmodium relictum*[41]	83
SW2	AF495572	*Plasmodium polare*[42]	9
GRW11	AY831748	*Plasmodium relictum*[43]	1
PARUS1	AF254977	*Haemoproteus majoris*[44]	2
PHSIB1	AF495565	*Haemoproteus majoris*[44]	2
multiple infection	—	*Plasmodium* sp. &*Haemoproteus * sp.	2
PARUS4	AY393795	*Leucocytozoon* sp.	11
PARUS16	—	*Leucocytozoon* sp.	4
PARUS18	—	*Leucocytozoon* sp.	1
PARUS19	—	*Leucocytozoon* sp.	16
PARUS22	—	*Leucocytozoon* sp.	16
PARUS25 *	JX855044	*Leucocytozoon* sp.	2
PARUS26 *	JX855045	*Leucocytozoon* sp.	2
PARUS28 *	JX855047	*Leucocytozoon* sp.	1
PARUS34 *	JX855049	*Leucocytozoon* sp.	1
PARUS35 *	JX855050	*Leucocytozoon* sp.	1
PARUS36 *	JX855051	*Leucocytozoon* sp.	1
PARUS37 *	JX855052	*Leucocytozoon* sp.	1
multiple infection	—	*Leucocytozoon* sp.	25

Number of observations (*n*) of*Plasmodium, Haemoproteus* and*Leucocytozoon* lineages across three years in great tit (Parus major) individuals. Lineages marked with an asterisk (*) have not previously been encountered in the literature.

**Table 2:** ***Leucocytozoon*lineages cloned from multiple infections**

	GenBank	Number of
Lineage	Accession no.	individuals infected
Lineages also involved in single infections (see Table 1):		
PARUS4	AY393795	2
PARUS16	—	3
PARUS19	—	2
PARUS22	—	5
Lineages involved in multiple infections only:		
PARUS29 *	JX855048	1

Leucocytozoon lineages cloned from a random subset of ten great tits (Parus major) showing multiple infections. Only lineages recorded in two or more different colonies per sample were considered. The lineage marked with an asterisk (*) has not previously been encountered in the literature.

### Co-infections

Fourty-four (81.5%) of the infected individuals showed co-infection with either *Plasmodium* or *Haemoproteus* and *Leucocytozoon* at the same time. Plasmodium parasitaemia was not affected by co-infection with *Leucocytozoon* (LRT: χ^2^ = 0.09, df = 1, p = 0.759) and body mass was not significantly affected by concurrent infection with *Plasmodium relictum* lineage SGS1 and Leucocytozoon (LRT: χ^2^ = 0.18, df = 1, p = 0.671).

## Discussion

This study examined haemosporidian infection at the individual level in wild free-living great tits across time. Parasite prevalence was high (98%) with only one individual remaining parasite free across both years of its capture. A co-infection rate of 82% was found in recaptured birds. While co-infection can influence parasite dynamics, no relationship was found for the presence of *Leucocytozoon* parasites and *Plasmodium* parasitaemia. The data indicates that *Leucocytozoon* infection is dynamic within a host and between several parasite lineages whilst on the other hand and in agreement with Hasselquist *et al*[25], Plasmodium and Haemoproteus infection remains constant once a parasite has established itself within a host.

### Parasite persistence

The differences observed in the present study with regard to *Plasmodium, Haemoproteus* and *Leucocytozoon* lineage turn-over within hosts might be an indication of the differential strategies applied by different parasites when infecting their hosts, and might be explained by two levels of interaction: within-host competition between parasite lineages and host immune defense.

*Leucocytozoon* parasites in the great tits might not be as effective at competing for resources as *Plasmodium* or *Haemoproteus* parasites, and as a result cannot increase in frequency to such an extent as to gain enough of a competitive advantage over Plasmodium parasites to be able to persist within the host’s blood in the presence of *Plasmodium*. This would not necessarily imply any transmission disadvantage for Leucocytozoon, which might choose to undertake larger-scale sexual reproduction, while Plasmodium infection consists of a globally larger number of asexual parasites. This possibility is illustrated by a study on rodent malaria (Plasmodium chabaudi) where Taylor et al [45] compared infection dynamics of two lineages when inoculated individually into hosts or as co-infections with different initial inoculation frequencies. This was done to test whether competitive interactions between parasites occur in terms of infection of hosts and transmission to vectors. The authors concluded that malaria parasites might have evolved to maximize transmission from mixed-genotype infections, as asexual abundance did not correlate with transmission success in co-infections. This would therefore imply that different parasites involved in co-infections might differentially invest in either asexual or sexual reproduction, thereby ensuring transmission potential to the parasite occurring in lower numbers in the presence of competition. This may, however, hold true only for mixed infection with lineages belonging to the same genus and which share the same vectors.

Another explanation for the differences in turn-over among lineages could be the host’s immune system. Hosts are not passive victims of parasite infections, but actively launch a defensive campaign in the form of the immune system (for a full review on the mechanisms by which haemosporidian parasites evade the host’s immune response see [46]). The immune components and defense strategies applied during infection will vary amongst different host species, especially with regard to inflammatory response and adaptive immunity (for a review, see [47]). Little information is available on the evolutionary history of *Leucocytozoon*. One can speculate that in this study system, the bird’s immune system might be more competent at fighting off Leucocytozoon infection than either *Plasmodium* or *Haemoproteus* infections, indirectly inferring a competitive advantage to the latter and resulting in greater lineage and infection status turn-over.

It must also be noted that the possibility exists that hosts might be able to clear parasites of all genera equally efficiently, but repeat infection probability of *Plasmodium* parasites (which occur in greater abundances than other parasites within the study population) is higher than for *Leucocytozoon* parasites which show a greater lineage diversity, but at lower prevalences. This could result in an artefact illusion that *Plasmodium* infection is more persistent. This possibility could be tested by serial sampling throughout the season.

### Lineage specific differences

The general life-cycle and process of Haemosporida infection are well-known and accepted as a whole [8], but very little is known about the exact infection dynamics within hosts. Parasitaemia experienced by great tits in this study was lineage dependent, with SW2 infection resulting in higher parasitaemia than SGS1. This is interesting, as Valkiūnas [8] reports that infection with P. polare, which is the morphospecies to which SW2 belongs, always results low parasitaemia. This observed discrepancy with the literature could point to sampling time coinciding with peak parasitaemia in SW2, but at this time no other explanation can be given. Such lineage-specific differences between parasites in parasitaemia as observed in this study is in concurrence with Zehtindjiev et al [48] who found that prepatent period, peak parasitaemia and parasitaemia during chronic infection differed by parasite lineage in great reed warblers (Acrocephalus arundinaceus). Comparably, Palinauskas et al [49] found that pre-patent period and peak parasitaemia also differed among firstly, different host species and secondly, individuals within the same species experimentally infected with the same parasite lineage. It therefore appears that infection dynamics are idiosyncratic and can depend on many factors and their interactions. One such confounding factor influencing infection dynamics is that hosts face the onslaught of many parasites at the same time, and rarely have to cope with single infections in nature, i.e. within-host competition as a result of mixed infections.

### Co-infection

The data from this study show that co-infections of malaria parasites are common, and our estimate in the great tit of 82% is consistent with the high frequencies of infection with multiple parasites in natural populations of other European bird species reported by Valkiūnas et al [50]. Despite this high prevalence of co-infections, there does not appear to be a negative effect of co-infection on Plasmodium parasitaemia. This could be evidence for a more synergistic/benign type of interaction as summarized in [33], where a parasite induces prolonged infection or better establishment of another parasite. In addition, the possibility that the latter could influence Leucocytozoon parasitaemia, cannot be excluded, as this was beyond the scope of this investigation. Another important consideration is that, while this study only considers a co-occurrence of parasites, effects might vary with the number of parasites involved in co-infections.

The large number of Leucocytozoon multiple infections observed, as opposed to multiple Plasmodium and Haemoproteus infections, might indicate Leucocytozoon parasites to be opportunistic parasites which can only establish themselves once a host’s immune system is weakened by prior infections thereby facilitating their establishment. This is in accordance with Cornet and Sorci [51] who investigated the virulence consequences of parasite-induced immunosuppression on the risk of contracting opportunistic diseases in Gammarus pulex. Their study suggests that parasite exploitation of the host depends on the risk of contracting opportunistic diseases. Parasites should therefore limit their suppression of the host’s immune response if opportunistic infections will result in host death, but should not have to change their exploitation if the risk associated therewith is low. Accordingly, opportunistic Leucocytozoon lineages might not impose significant virulence costs to their hosts so as to allow for infection opportunity. As Leucocytozoon parasitaemia was not tested in this study, it is impossible to know if multiple Leucocytozoon infections result in higher parasitaemia than single infections and is a definite avenue for future investigation. If this is a case of facilitated, opportunistic infection, it would be evidence that not all lineages have equal infection potential.

## Conclusion

Most studies only consider Plasmodium and Haemoproteus parasites when investigating avian haemosporidian infections and as a result Leucocytozoon is under-represented in the literature. This study has focussed not only on Plasmodium, Haemoproteus and Leucocytozoon infection across time at the individual scale, but also considered multiple infections of these parasites and what that might imply for Haemosporida coevolution with their hosts. This study has demonstrated the complexities of interactions (host-parasite and parasite-parasite) and dynamics of parasite communities and their hosts in natural study systems which could affect the ecology of either or both organisms involved.

## Competing interests

The authors declare that they have no competing interests.

## Authors’ contributions

PC and OG participated in the design of the study, assisted with data collection and manuscript drafting. FL contributed to data collection and analyses. JR collected data, performed molecular analyses, statistical analyses and drafted the manuscript. All authors read and approved the final manuscript.

## References

[B1] World Health Organization World Malaria Report 2012 2012

[B2] Bensch S Hellgren O Pérez-Tris J MalAvi: a public database of malaria parasites and related haemosporidians in avian hosts based on mitochondrial cytochrome b lineages Mol Ecol Res 2009 9 1353 1358 10.1111/j.1755-0998.2009.02692.x 21564906

[B3] Valkiūnas G Zickus T Shapoval AP Iezhova TA Effect of Haemoproteus belopolskyi (Haemosporida: Haemoproteidae) on body mass of the blackcap Sylvia atricapilla J Parasitol 2006 92 1123 1125 10.1645/GE-3564-RN.1 17152968

[B4] Merino S Moreno J Sanz JJ Arriero E Are avian blood parasites pathogenic in the wild? A medication experiment in blue tits (Parus caeruleus) Proc R Soc B 2000 267 2507 2510 10.1098/rspb.2000.1312 11197126 PMC1690848

[B5] Marzal A de Lope F Navarro C Møller AP Malarial parasites decrease reproductive success: an experimental study in a passerine bird Oecologia 2005 142 541 545 10.1007/s00442-004-1757-2 15688214

[B6] Tomás G Merino S Moreno J Morales J Martínez-de la Puente J Impact of blood parasites on immunoglobulin level and parental effort: a medication field experiment on a wild passerine Funct Ecol 2007 21 125 133

[B7] Dawson RD Bortolotti GR Effects of hematozoan parasites on condition and return rates of American Kestrels Auk 2000 117 373 380

[B8] Valkiūnas G Avian Malaria Parasites and Other Haemosporidia 2005 Boca Raton: CRC Press

[B9] Navarro C de Lope F Marzal A Møller AP Predation risk, host immune response, and parasitism Behav Ecol 2004 15 629 635 10.1093/beheco/arh054

[B10] Møller AP Nielsen JT Malaria and risk of predation: A comparative study of birds Ecology 2007 88 871 881 10.1890/06-0747 17536704

[B11] Van Riper C Van Riper SG Goff ML Laird M The epizootiology and ecological significance of malaria in Hawaiian landbirds Ecol Monogr 1986 56 327 344 10.2307/1942550

[B12] Atkinson CT Woods KL Dusek RJ Sileo LS Iko WM Wildlife disease and conservation in Hawaii: pathogenicity of avian malaria (Plasmodium relictum) in experimentally infected ‘L’iwi (Vestiaria coccinea) Parasitology 1995 111 Suppl S59—S69 8632925 10.1017/s003118200007582x

[B13] Atkinson CT Dusek RJ Woods KL Iko WM Pathogenicity of avian malaria in experimentally-infected Hawaii Amakihi J Wildl Dis 2000 36 197 204 10813599 10.7589/0090-3558-36.2.197

[B14] Bensch S Stjernman M Hasselquist D Östman O Hansson B Westerdahl H Pinheiro RT Host specificity in avian blood parasites: a study of Plasmodium and Haemoproteus mitochondrial DNA amplified from birds Proc R Soc B 2000 267 1583 1589 10.1098/rspb.2000.1181 11007335 PMC1690711

[B15] Waldenström J Bensch S Kiboi S Hasselquist D Ottosson U Cross-species infection of blood parasites between resident and migratory songbirds in Africa Mol Ecol 2002 11 1545 1554 10.1046/j.1365-294X.2002.01523.x 12144673

[B16] Scheuerlein A Ricklefs RE Prevalence of blood parasites in European passeriform birds Proc R Soc B 2004 271 1363 1370 10.1098/rspb.2004.2726 15306334 PMC1691737

[B17] Ortego J Calabuig G Cordero PJ Aparicio JM Genetic characterization of avian malaria (Protozoa) in the endangered lesser kestrel, Falco naumanni Parasitol Res 2007 101 1153 1156 10.1007/s00436-007-0575-y 17514379

[B18] Wiersch SC Lubjuhn T Maier WA Kampen H Haemosporidian infection in passerine birds from Lower Saxony J Ornithol 2007 148 17 24

[B19] Krone O Waldenström J Valkiūnas G Lessow O Müller K Iezhova TA Fickel J Bensch S Haemosporidian blood parasites in European birds of prey and owls J Parasitol 2008 94 709 715 18605786 10.1645/GE-1357.1

[B20] Jenkins T Owens IPF Biogeography of avian blood parasites (Leucocytozoon spp.) in two resident hosts across Europe: phylogeographic structuring or the abundance-occupancy relationship? Mol Ecol 2011 20 3910 3920 10.1111/j.1365-294X.2011.05221.x 21880082

[B21] Glaizot O Fumagalli L Iritano K Lalubin F Van Rooyen J Christe P High prevalence and lineage diversity of avian malaria in wild populations of great tits (Parus major) and mosquitoes (Culex pipiens) PLoS ONE 2012 7 e34964 10.1371/journal.pone.0034964 22506060 PMC3323596

[B22] Bensch S Waldenström J Jonzén N Westerdahl H Hansson B Sejberg D Hasselquist D Temporal dynamics and diversity of avian malaria parasites in a single host species J Anim Ecol 2007 76 112 122 10.1111/j.1365-2656.2006.01176.x 17184359

[B23] Fallon SM Ricklefs RE Latta SC Bermingham E Temporal stability of insular avian malarial parasite communities Proc R Soc B 2004 271 493 500 10.1098/rspb.2003.2621 15129959 PMC1691613

[B24] Spurgin LG Illera JC Padilla DP Richardson DS Biogeographical patterns and co-occurrence of pathogenic infection across island populations of Berthelot’s pipit (Anthus berthelotii) Oecologia 2012 168 691 701 10.1007/s00442-011-2149-z 21983713

[B25] Hasselquist D Östman O Waldenström J Bensch S Temporal patterns of occurrence and transmission of the blood parasite Haemoproteus payevskyi in the great reed warbler Acrocephalus arundinaceus J Ornithol 2007 148 401 409 10.1007/s10336-007-0144-2

[B26] Knowles SCL Wood MJ Alves R Wilkin TA Bensch S Sheldon BC Molecular epidemiology of malaria prevalence and parasitaemia in a wild bird population Mol Ecol 2011 20 1062 1076 10.1111/j.1365-294X.2010.04909.x 21073677

[B27] Piersma T Van der Velde M Dutch House Martins Delichon urbicum gain blood parasite infections over their lifetime, but do not seem to suffer J Ornithol 2012 153 907 912 10.1007/s10336-012-0826-2

[B28] Latta SC Ricklefs RE Prevalence patterns of avian haemosporida on Hispaniola J Avian Biol 2010 41 25 33 10.1111/j.1600-048X.2009.04685.x

[B29] Roulin A Christe P Dijkstra C Ducrest AL Jungi TW Origin-related, environmental, sex, and age determinants of immunocompetence, susceptibility to ectoparasites, and disease symptoms in the barn owl Biol J Linn Soc 2007 90 703 718 10.1111/j.1095-8312.2007.00759.x

[B30] Doolan DL Dobaño C Baird JK Acquired Immunity to Malaria Clin Microbiol Rev 2009 22 13 36 10.1128/CMR.00025-08 19136431 PMC2620631

[B31] Christe P Giorgi MS Vogel P Arlettaz R Differential species-specific ectoparasitic mite intensities in two intimately coexisting sibling bat species: resource-mediated host attractiveness or parasite specialization? J Anim Ecol 2003 72 866 872 10.1046/j.1365-2656.2003.00759.x

[B32] de Roode JC Pansini R Cheesman SJ Helinski MEH Huijben S Wargo AR Bell AS Chan BHK Walliker D Read AF Virulence and competitive ability in genetically diverse malaria infections PNAS 2005 102 7624 7628 10.1073/pnas.0500078102 15894623 PMC1140419

[B33] Palinauskas V Valkiūnas G Bolshakov CV Bensch S Plasmodium relictum (lineage SGS1) and Plasmodium ashfordi (lineage GRW2): The effects of the co-infection on experimentally infected passerine birds Exp Parasitol 2011 127 527 33 10.1016/j.exppara.2010.10.007 21050849

[B34] Marzal A Bensch S Reviriego M Balbontin J De Lope F Effects of malaria double infection in birds: one plus one is not two J Evol Biol 2008 21 979 987 10.1111/j.1420-9101.2008.01545.x 18462316

[B35] Davidar P Morton ES Are multiple infections more severe for Purple Martins (Progne subis) than single infections? Auk 2006 123 141 147 10.1642/0004-8038(2006)123[0141:AMIMSF]2.0.CO;2

[B36] Waldenström J Bensch S Hasselquist D Östman O A new nested polymerase chain reaction method very efficient in detecting Plasmodium and Haemoproteus infections from avian blood J Parasitol 2004 90 191 194 10.1645/GE-3221RN 15040694

[B37] Christe P Glaizot O Strepparava N Devevey G Fumagalli L Twofold cost of reproduction: an increase in parental effort leads to higher malarial parasitaemia and to a decrease in resistance to oxidative stress Proc R Soc B 2012 279 1142 1149 10.1098/rspb.2011.1546 21920974 PMC3267143

[B38] Hellgren O Waldenström J Bensch S A new PCR assay for simultaneous studies of Leucocytozoon, Plasmodium, and Haemoproteus from avian blood J Parasitol 2004 90 797 802 10.1645/GE-184R1 15357072

[B39] R-Cran Project, version 2.15.0 [http://www.R-project.org]

[B40] Bates DM Sarkar D lme4: Linear mixed-effects models using S4 classes. R package version 0.99875-6 2007

[B41] Palinauskas V Kosarev V Shapoval A Bensch S Valkiūnas GN Comparison of mitochondrial cytochrome b lineages and morphospecies of two avian malaria parasites of the subgenera Haemamoeba and Giovannolaia (Haemosporida: Plasmodiidae) Zootaxa 2007 1626 39 50

[B42] Beadell JS Ishtiaq F Covas R Melo M Warren BH Atkinson CT Bensch S Graves GR Jhala YV Peirce MA Rahmani AR Fonseca DM Fleischer RC Global phylogeographic limits of Hawaii’s avian malaria Proc R Soc B 2006 237 2953 2944 10.1098/rspb.2006.3671 PMC1639517 17015360

[B43] Valkiūnas G Zehtindjiev P Dimitrov D Križanauskiene A Iezhova TA Bensch S Polymerase chain reaction-based identification of Plasmodium (Huffia) elongatum, with remarks on species identity of haemosporidian lineages deposited in GenBank Parasitol Res 2008 102 1185 1193 10.1007/s00436-008-0892-9 18270739

[B44] Križanauskiene A Hellgren O Kosarev V Sokolov L Bensch S Valkiūnas G Variation in host specificity between species of avian haemosporidian parasites: Evidence from parasite morphology and cyochrome b gene sequences J Parasitol 2006 92 1319 1324 10.1645/GE-873R.1 17304814

[B45] Taylor LH Walliker D Read AF Mixed-genotype infections of malaria parasites: within-host dynamics and transmission success of competing clones Proc R Soc B 1997 264 927 935 10.1098/rspb.1997.0128 9225482 PMC1688430

[B46] Zambrano-Villa S Rosales-Borjas D Carrero JC Ortiz-Ortiz L How protozoan parasites evade the immune response Trends Parasitol 2002 18 272 278 10.1016/S1471-4922(02)02289-4 12036742

[B47] Hasselquist D Comparative immunoecology in birds: hypotheses and tests J Ornithol 2007 148 S571—S582

[B48] Zehtindjiev P Ilieva M Westerdahl H Hansson B Valkiūnas G Bensch S Dynamics of parasitemia of malaria parasites in a naturally and experimentally infected migratory songbird, the great reed warbler Acrocephalus arundinaceus Exp Parasitol 2008 119 99 110 10.1016/j.exppara.2007.12.018 18280472

[B49] Palinauskas V Valkiūnas GN Bolshakov CV Bensch S Plasmodium relictum (lineage P-SGS1): Effects on experimentally infected passerine birds Exp Parasitol 2008 120 372 380 10.1016/j.exppara.2008.09.001 18809402

[B50] Valkiūnas G Iezhova TA Shapoval AP High prevalence of blood parasites in hawfinch Coccothraustes coccothraustes J Nat Hist 2003 37 2647 2652 10.1080/002229302100001033221

[B51] Cornet S Sorci G Parasite virulence when the infection reduces the host immune response Proc R Soc B 2010 277 1929 1935 10.1098/rspb.2010.0138 20200031 PMC2871884

